# Social ecological factors and intimate partner violence in pregnancy

**DOI:** 10.1371/journal.pone.0194681

**Published:** 2018-03-29

**Authors:** Bosena Tebeje Gashaw, Berit Schei, Jeanette H. Magnus

**Affiliations:** 1 College of Health Sciences, Jimma University, Jimma, Ethiopia; 2 Faculty of Medicine, University of Oslo, Oslo, Norway; 3 Department of Public Health and Nursing, Faculty of Medicine and Health Sciences, University of Science and Technology, Trondheim, Norway; 4 Department of Obstetrics and Gynaecology, St. Olav’s hospital, Trondheim University Hospital, Trondheim, Norway; 5 Department of Global Community Health and Behavioral Sciences, Tulane School of Public Health and Tropical Medicine, New Orleans, Louisiana, United States of America; King's College London, UNITED KINGDOM

## Abstract

**Background:**

Intimate partner violence (IPV) during pregnancy increases adverse pregnancy outcomes. Knowledge of societal, community, family and individual related factors associated with IPV in pregnancy is limited in Ethiopia. Our study examined these factors in an Ethiopian context.

**Materials and methods:**

A cross sectional study was conducted among pregnant women attending antenatal care at governmental health institutions, using a consecutive probability sampling strategy. A total of 720 pregnant women were interviewed by five trained nurses or midwives, using a standardized and /pretested survey questionnaire. Bivariate and multivariate logistic regression analyses were applied to assess factors contributing to IPV. We used Akaike’s information criteria, to identify the model that best describes the factors influencing IPV in pregnancy.

**Results:**

Among the women interviewed, physical IPV was reported by 35.6%, and lifetime emotional or physical abuse by 81.0%. Perceiving violence as a means to settle interpersonal conflicts, presence of supportive attitudes of wife beating in the society, regarding violence as an expression of masculinity, and presence of strict gender role differences in the society, were all positively associated to IPV in pregnancy. The presence of groups legitimizing men’s violence in the community, feeling isolated, having no social support for victims, and presence of high unemployment, were the perceived community related factors positively associated with IPV in pregnancy.

**Conclusion:**

IPV in pregnancy is very prevalent in Ethiopia and is associated with multiple social ecologic factors. Reduction of IPV in pregnancy calls for cross sectorial efforts from stakeholders at different levels.

## Background

Intimate partner violence (IPV), is an important global public health and human rights issue, with significant health and socioeconomic development consequences [1]. Violence against women may occur at any stage of a woman’s life, including during pregnancy. The overall global estimates of IPV around the time of pregnancy vary between 3–30% [2], with higher prevalence reported in developing countries [3]. In Africa, the prevalence of pregnancy related IPV is reported to be between 23–40% [4]. Prior studies in Ethiopia indicate a very high life time prevalence of IPV among reproductive age women at 50–78% [5–10], and pregnancy related IPV between 11–29% [11–15]. The majority of these studies examined factors contributing to IPV in pregnancy related to the individual context, such as childhood inter-parental exposure, early marriage, dowry payment, residence, alcohol use and or education. *It is important to* conceptualize violence as a multifaceted phenomenon grounded in the interplay across societal, community, family, and individual levels. There is a lack of studies that simultaneously examine societal, community, family, and individual related factors contributing to IPV in pregnancy in Africa and in Ethiopia in particular.

A healthy pregnancy is required for favourable maternal and child health outcomes. Pregnancies affected by IPV are reported to have an increased incidence of low maternal weight gain, anaemia, infection, first/second trimester bleeding, late entry into antenatal care (ANC), preterm labour, premature birth and low birth weight baby [16, 17]. Homicide is also one of the leading causes of pregnancy associated death, commonly as a consequence of IPV [18]. IPV may commence or escalate in pregnancy [19]. Studies report that IPV during pregnancy is more common than some maternal health conditions routinely screened for during antenatal care [4, 20]. The causes for IPV are complex and dependent on the context [21]. Many of the previous studies have identified various individual and family related risk factors [11, 13, 15, 22, 23]. Few empirical studies have, however, explored the association of all social ecologic factors, (specifically societal and community) with IPV in pregnancy.

This study aimed to address the gaps in the existing literature concerning the social ecologic factors that make pregnant women vulnerable to IPV. Guided by the social ecological model (SEM), the current study examined the prevalence, pattern and the association between social ecologic factors (related to the society, community, family and individual) and IPV in pregnancy in an Ethiopian context.

## Materials and methods

### Study design and population

This cross sectional study was conducted during an antenatal care visit at all health centres, and hospitals in Jimma, 177, 900 inhabitants, (CSA, 2015) and, located in Oromiya regional state, 352 km south west of Addis Ababa, Ethiopia, from November 2015 to March 30, 2016.

Included were women, with a pregnancy estimated to be ≥ 24 weeks of gestation. Sample size was calculated based on a single population proportion formula using the assumptions of: 95% confidence interval, 4% degree of precision. We assumed 50% as the expected prevalence based on the average from previous studies [4, 11] and a 20% non-response rate for our power calculation. The estimated number of women was proportionally recruited in all study facilities based on their average monthly client flow. The proportional allocation ranged from 78–233.

### Data collection tools and strategy

A standard questionnaire was developed based on the Abuse Assessment Screening (AAS) tools developed by the Nursing Research Consortium on Violence [24–26] and the social ecologic factors adapted from the 2005 WHO practical guidelines for researchers [27, 28]. The compiled questionnaire was translated from English language to Amharic and Afan Oromo by a translator and back-translated to English language by a second translator to ensure consistency. Pre-test covered 5% of the sampled pregnant women with similar socio demographic characteristics using institutions not included in the study. Minor modifications were made to the AAS tool to capture the different types of IPV in behavioural terms.

The study data were collected by five female, midwives/nurses working at the respective institutions, fluent in both languages (Afan Oromo and Amharic). Training was given on how to interview, handling ethical issues and maintaining confidentiality and privacy using a training manual. During data collection, to prevent incomplete and inconsistent responses, the researcher and supervisors were available for supervising and counter checking completed questionnaires.

### Measurements and data analysis

The experience of Intimate Partner Violence (S2 File) was measured using Abuse Assessment Screening (AAS) tools: to measure the lifetime emotional or physical violence, women were asked if they have ever been emotionally or physically abused by their partner or someone important to them, with a response of Yes/No; Within the last year physical violence was measured, if women have ever been hit, slapped, kicked, or otherwise physically hurted by someone within the last year, response, Yes/No; if Yes, who? (Husband, Ex-husband, Boy friend, Stranger, In-laws, Multiple); Physical violence in the current pregnancy was measured, whether women have been slapped, kicked, or otherwise physically hurted by someone during the current pregnancy, response, Yes/No); If Yes, who? (Husband, Ex-husband, Boyfriend, Stranger, In-laws, Multiple); again If Yes, where? (on the Face, Head, Abdomen, Back, Buttock, Other, state); Incidents of physical violence were scored according to the following scale: (1 = Threats of abuse including use of weapon, 2 = Slapping, pushing with no injuries and/or lasting pain, 3 = Punching, kicking, bruises, cuts and/or continuing pain, 4 = Beating up, severe contusions, burns or broken bones, 5 = Head injury, internal injury or permanent injury, 6 = Use of weapon (gun, knife), or wound from weapon); Within the last year sexual violence was measured, if women have been forced to have sexual activities by anyone within the last year, response, Yes/No, If Yes, who? (Husband, Ex-husband, Boy friend, Stranger, Multiple); Whether the women were afraid of their partner or anyone listed above, response, Yes/No. Women were also asked where did they turn in the incidents of any IPV, response: their family, neighbors, religious father, keep silent, or other state; If they keep silent, they were asked to state major reason/s. Additional tools that were not included in the AAS in behavioral terms were added to measure psychological violence [i.e, within the last year, if women have been insulted, belittled, constantly humiliated, intimidated (e.g. destroying things), threatened of being harmed and/ threatened to take away children by their partner]; and controlling behavior which was measured, whether women have been controlled by their partner with in the last year, with the response of any of the following: isolated from family and friends, monitored their movements, restricted access to financial resources, restricted from employment, education or medical care.

IPV during the current pregnancy was the dependent variable; operationalized in this study as, answering ‘Yes’ to any of the following: “since you’ve been pregnant (current pregnancy), have you been slapped, kicked, or otherwise physically hurted by your intimate partner (husband, ex-husband or boyfriend)”. While, each item under the four domains of social ecologic model (Societal domain, Community domain, Family domain and Individual domain) were considered as exposure variables and their responses were coded as Yes/No for each.

All information were entered in EPiData and exported to SPSS (version 20.0) for analysis.

Multi- collinearity or redundancy, [29], was checked by variance inflation factor, tolerance test and the standard errors of the regression coefficients. Consistency (internal validity) of factors under each domain were measured by Cronbach’s alpha and were all 0.7 or above. Descriptive statistics, such as frequency, percent, mean with (SD) and median were computed to summarize baseline characteristics. Comparative analyses using chi-square p-value test was done to explore association between various socio-demographic characteristics of the pregnant women and her partner with IPV in pregnancy (Table 1). Both univariate and multivariate logistic regression models were used to assess the unadjusted and adjusted association, respectively. Only significant variables in the crude analysis were entered into the multivariate logistic regression analysis. Potential confounders (age and education of the woman and her partner) were considered based on their significant statistical association between their effects (both to exposure and outcome) in the crude analyses and based on the findings in earlier studies [10, 30]. In the multivariable logistic regression analysis 95% confidence interval (CI) for OR (odds ratio) was calculated. Backward elimination variable selection process for multiple regression was used to identify the final significant and independent variables [31].

**Table 1:** Socio-demographic characteristics of pregnant women and their partner attending antenatal care, Jimma, Ethiopia, (N = 720).

Variables	All women (720) No. (%)	IPV in current pregnancy		P-value
		Yes (256) No. (%)	No (464) No. (%)	
**Woman**				
**Age**				
15–24	344(47.8)	104(30.2)	240(69.8)	**<0.001**
25–34	334(46.4)	125(37.4)	209(62.6)	
35–45	42(5.8)	27(64.3)	15(35.7)	
**Relationship to current partner**				
Married	609(84.6)	226(37.1)	383(62.9)	NS
Cohabited	100 (13.9)	25(25.0)	75(75.0)	
Boy Friend	11(1.5)	5(45.6)	6(54.5)	
**Ethnicity**				
Oromo	421(58.5)	160 (38.0)	261(62.0)	NS
Amhara	105(14.6)	33(31.4)	72(68.6)	
Guragie	50(6.9)	18(36.0)	32(64.0)	
Kefa	61(8.5)	15(24.6)	46(75.4)	
Dawro	53(7.4)	18(34.0)	35(66.0)	
other (Yem, Tigre, Wolayita)	30(4.2)	12(40.0)	18(60.0)	
**Childhood residence**				
Urban	250(34.7)	58(23.2)	192(76.8)	**<0.001**
Rural	470(65.3)	198(42.1)	272(57.9)	
**Religion**				
Orthodox	219(30.4)	70(32.0)	149 (68.0)	NS
Muslim	407(56.5)	157(38.6)	250 (61.4)	
Protestant and Catholic	94(13)	29(30.9)	65 (69.1)	
**Marital duration**				
less than 1 year	135(18.8)	26(19.3)	109(80.7)	**<0.001**
2–4 years	241(33.5)	73(30.3)	168(69.7)	
5–10 years	239(33.2)	108 (45.2)	131(54.8)	
More than 10 years	105(14.6)	49 (46.7)	56 (53.3)	
**Educational status**				
Read and write or no education	230(31.9)	125(54.3)	105(45.7)	**<0.001**
Primary cycle (1–8)	227(31.6)	75 (33.0)	152 (67.0)	
Secondary and above	263(36.5)	56 (21.3)	207(78.7)	
**Generate income by yourself**				
Yes	296(41.1)	95(32.1)	201(67.9)	NS
No	424(58.9)	161(38.0)	263(62.0)	
**Partner**				
**Age**				
20–35	446(61.9)	130(29.4)	316(70.6)	**<0.001**
35 and above	274(38.1)	126(46.0)	148(54.0)	
**Monthly income**				
< = 500	67(9.3)	28(41.8)	39(58.2)	NS
501–1000	164(22.8)	60(36.6)	104(63.4)	
1001–1500	112(15.6)	49(43.7)	63(56.3)	
> 1500	377(52.4)	119(31.6)	258(68.4)	
**Educational status**				
Read and write or no education	192(26.7)	106(55.2)	86(44.8)	**<0.001**
Primary cycle (1–8)	190(26.4)	68(35.8)	122(64.2)	
Secondary and above	338(46.9)	82(24.3)	256(75.7)	
**Occupation**				
Governmental employee	160(22.2)	42(26.3)	118(73.7)	**<0.001**
Private business or Merchant	346(48.0)	108 (31.2)	238(68.8)	
Farmer	148(20.6)	85 (57.4)	63 (42.6)	
Other (pensioner, driver, daily laborer, religious leader)	66(9.2)	21 (31.8)	45 (68.2)	

We used Akaike’s information criteria (AIC), [32–36] and the principles of parsimony (simple model) with few covariates, as results based on such a model promote numerical stability and generalizability of the results [32]; to identify the model that offered the best estimate of our data explaining the outcome, while bearing in mind that there is no single best model. AIC is increasingly being used when the analysis explore a range of variables associated with a particular behaviour [32–34], and used for estimating the predictive accuracy of models and guards against over fitting penalty. Based on AIC, a model with less AIC means better fit and explains the outcome. Burnham and Anderson in 2002 also noted, once the most parsimonious model is established, the traditional null-hypotheses testing can be used to make a statistical inference [37].

While adjusting for confounders (age and education of the woman and her partner), we fitted the model of each single factor, with all variables of each domain simultaneously and within the variables under each domain. After analyzing the adjusted OR, and considering the over fitting, under fitting and parsimony [36, 37], the final decision on how to interpret the result was made (Table 2). Finally (Table 3), a composite score (sum of items under each areas /domain factors) based on the frequency of individual Yes/No (Yes = 1; No = 0) responses was created. Not anticipating a normal distribution, those scoring above the median score were classified as having a positive value. While keeping the above model selection principles, the dichotomized four domain factors (societal, community, family and individual) were entered simultaneously into multivariate logistic regression model, adjusted for age and educational level of the woman and partner to analyze the strength of the association and independent effect of each domain with IPV in pregnancy.

**Table 2:** The association (crude and adjusted odds ratio (OR) of societal, community, family and individual domain factors with IPV in pregnancy (n = 720).

Social Ecological factors	IPV in current pregnancy		Crude OR, (95% CI)	Adjusted OR, (95% CI) Model I*	Adjusted OR, (95% CI) Model II **
	Yes (256) No. (%)	No (464) No. (%)			
**Societal**					
Presence of strict traditional gender norms in the society (ex, women should stay at home)	235(44.0)	299(56.0)	6.2 (3.8,10.0)	5.2 (3.2, 8.6)	NS
Violence is used as a means to settle interpersonal conflicts in the society	184(57.0)	139(43.0)	6.0 (4.3,8.4)	5.1 (3.6, 7.2)	2.6 (1.7,3.9)
Violence towards women is accepted in the society	207(51.5)	195(48.5)	6.0 (4.1,8.4)	5.0 (3.4,7.3)	NS
The society has supportive attitudes of wife beating	197(57.1)	148(42.9)	7.1 (5.0,10.1)	6.3 (4.4, 9.1)	2.6 (1.7,4.2)
The society regards violence as a notion of masculinity. (ex, a real man disciplines his wife)	207(50.7)	201(49.3)	5.5 (3.9,7.9)	4.8 (3.3,7.0)	1.8 (1.2,2.9)
There are strict gender role differences between the two sexes in the society (ex. Cooking is a women’s responsibility)	242(42.7)	325(57.3)	7.4 (4.2,13.1)	6.6 (3.6,11.8)	4.1 (2.2,7.8)
**Community**					
There are men’s group that condone or legitimize men’s violence in her community	193(58.1)	139(41.9)	6.9 (4.9,9.7)	6.1 (4.3,8.8)	3.0(2.0,4.5)
Woman feel isolated from her community	158(55.2)	128(44.8)	4.2 (3.1,5.9)	3.8 (2.7,5.3)	2.5 (1.7,3.7)
Women are living in a community where there is no social support for victims of violence	191(56.8)	145(43.2)	6.5(4.6,9.1)	5.7 (4.0,8.1)	2.6(1.7,4.0)
Most of the members of her community have low socioeconomic status or suffer financial problems	236 (45.0)	289 (55.0)	7.2 (4.4,11.7)	5.5 (3.3,9.2)	NS
There are many unemployed people in your community	239(43.7)	308(56.3)	7.1 (4.2,12.1)	6.3 (3.6,10.8)	3.0 (1.6,6.0)
**Family/relation**					
Woman consider that she has marital conflict	232(95.9)	10(4.1)	24.8 (15.6,39.6)	21.3 (13.2,34.3)	6.7(3.9,11.5)
Household property/wealth controlled by a partner (ex, a car, money, cattle…)	210(63.4)	121(36.6)	12.9 (8.9,18.9)	10.8 (7.3,16.0)	2.4 (1.3,4.5)
Communication is difficult with her partner	199(72.6)	75(27.4)	18.1 (12.3,26.6)	15.0(10.1,22.3)	4.1 (2.6, 6.6)
Decision making in the family controlled by her partner.	205(60.7)	133(39.3)	1.5 (1.2,1.9)	8.6 (5.9,12.6)	NS
**Individual**					
Woman was abused in her childhood	232(50.8)	225(49.2)	10.3(6.5,16.2)	11.8 (7.3,19.2)	3.2 (1.7,6.1)
Woman witness marital violence in her childhood	241(47.5)	266(52.5)	12.0(6.9,20.8)	12.5(7.1,22.0)	NS
Woman had rejecting father in her childhood	45(52.9)	40(47.1)	2.3(1.4,3.6)	2.0(1.2,3.2)	NS
Partner experienced violence as a child	230(63.9)	130(36.1)	22.7 (14.4, 35.8)	21.0(13.1,33.4)	4.0 (2.0,8.2)
Partner witnessed violence as a child	226(68.4)	148(31.6)	16.1(10.5, 24.7)	16.5 (10.5,26.0)	4.0 (2.0,8.2)
Partner had a rejecting father	48 (55.8)	38 (44.2)	2.6(1.6,4.1)	2.3 (1.4,3.7)	NS
Partner use substances (Khat/ smoking/ alcohol or all)	238 (47.8)	260 (52.2)	10.4 (6.2,17.3)	9.3(5.5,15.7)	6.0 (3.2,11.3)
Partner has mental health, or personality problems (ex, anxiety, sadness, social isolation..)	163 (81.9)	36 (18.1)	20.8 (13.6, 31.9)	18.3 (11.8,28.4)	11.7 (6.8,20.2)

Reference category is—‘No’ for all predictors

* Each single variable, adjusted for–age and education level of the woman and her partner

** Variables under each domain, adjusted for- age and education level of the woman and her partner; CI-confidence interval

**Table 3:** Bivariate and multivariate logistic regression analysis of dichotomized composite scores of each social ecological domain factor associated with IPV in pregnancy (n = 720).

Factors	IPV in pregnancy		Crude OR, (95% CI)	Adjusted OR*, (95% CI)
**Societal factor**	Yes (256) No. (%)	No (464) No. (%)		
Yes	196(63.6)	112(36.4)	10.3 (7.2, 14.7)	2.1 (1.2,3.7)
No	60(14.6)	352(85.4)	1	1
**Community factor**				
Yes	186(69.7)	81(30.3)	12.6 (8.7, 18.1)	2.1 (1.2,3.6)
No	70(15.5)	383(84.5)	1	1
**Family factor**				
Yes	203(75.2)	67(24.8)	22.7 (15.2, 33.8)	5.8 (3.5,9.5)
No	53(11.8)	397(88.2)	1	1
**Individual factor**				
Yes	236(66.9)	117(33.1)	35.0 (21.2, 57.8)	12.2 (7.0,21.5)
No	20(5.4)	347(94.6)	1	1

* Adjusted for age and education level of the woman and her partner

CI: confidence interval

While keeping in mind the AIC, parsimony and under fit criteria of the best model, multivariate logistic regression model was fitted to examine significant variables of the four social ecologic domain factors associated with IPV in pregnancy (Table 3). The dichotomised sum of score for each domain of societal, community, family and individual factors were significantly and independently associated with IPV in pregnancy, adjusted for age and education of woman and partner.

### Ethical consideration

Ethical clearance from Jimma University College of Health Sciences, IRB (Ref No: HRPGC/305/2015) and the Norwegian Regional Ethical Committee (REK), [(Ref No: 2015/623 / REK Nord), (S1 File)] was obtained. Both of the ethical committees approved the verbal and written consent procedures. Data collection was in accordance with the recommendations of WHO Ethical Standard on Ethical and Safety Recommendations for Domestic Violence Research (WHO, 1999).

Informed written and or / verbal consent was obtained after fully informing participants about the aims and the nature of the study. Respondents were assured about the confidentiality of their response, their voluntarily participation and right to terminate at any time they want. They were also told that their care would not be affected in any way should they decide not to participate in the study.

## Results

### Socio-demographic characteristics of the pregnant women

Three hundred and forty four (47.8%) of the 720 participants, mean age SD (24.9±5.0), were between15-24 years and 34.4% were first time pregnant (primi gravida) (Table 1). Over one third had been married before the age of 18 years and in an arranged marriage. The majority of the women were the sole wife (85.1%) (data not shown). One of four women reported having no education, and only 41.1% of the women reported generating income and the majority earned less than 1000 birr/month ($45/month).

### The prevalence of IPV in pregnancy

In the current pregnancy, more than one in three women (35.6%) reported experiencing IPV, of which nearly 89% of them had experienced moderate physical violence (severity score of 1–3), slapping or pushing being the most frequent incidents (62.1%) and 18% reported being hit on their abdomen (data not shown). The life time prevalence of emotional or physical abuse by partner or someone important was 80.7% and the proportion of violence during pregnancy among ever exposed was 44%. Over half of the women (56.1%) reported physical violence victimization within the last year and the majority of the perpetrators (86.4%) were their husbands. Forced sexual activity was reported by half of the women, but only one woman was offended by a stranger.

Being isolated by intimate partner from friends and family was prevalent (40%) and more than half of the women reported fear (53.8%) or psychological abuse (51.4%) in the last one year. During incidents of IPV one in three turned to their family (32.4%) or kept silent (29.5%). The major reasons to keep silent were: not to leave children behind, feeling shame, to honour the tradition that marriage is valued, having no money and nowhere to go, and fear that husband may retaliate or even kill them (data not shown).

Using AIC and parsimony criteria of best model as described in the analysis section, the statistical inferences were made based on the traditional null-hypothesis testing (Table 2, Model II). A multivariate logistic regression model was fitted to examine significant variables under each social ecologic domain associated with IPV in pregnancy adjusted for age and education of woman and partner. Perceiving violence as a means to settle interpersonal conflicts, presence of supportive attitudes of wife beating in the society, regarding violence as an expression of masculinity, and presence of strict gender role differences in the society were significantly positively associated with IPV in pregnancy. Community related factors that significantly and positively associated with IPV in pregnancy included: the presence of groups legitimizing men’s violence, feeling isolated, having no social support for victims and the presence of high unemployment.

## Discussion

To our knowledge, the current study is the first in Ethiopia, if not in Africa, to explore the association of societal, community, family and individual related factors with IPV in pregnancy simultaneously. It also examined the prevalence of IPV in pregnancy in Ethiopia. The result indicated an increased prevalence of IPV associated with several factors. In recognition that no single factor could explain the complexity of why some women are at a higher risk of IPV while others are not [38, 39], we used the Social Ecologic Model, the most widely used model for understanding violence [28, 40]. Societal and community factors exhibit a strong and consistent influence on the occurrence of IPV in pregnancy coinsiding with a host of earlier reported family and individual risk factors [11–15].

Women perceiving that violence is used as a means to settle interpersonal conflicts in the society had a higher likelihood of IPV in pregnancy in this study. Earlier studies have also discussed that when societies endorse violence as a strategy in conflict resolution, and some levels of conflicts are likely in marriage [21, 28, 41], makes it challenging to reject IPV. In the present study, supportive attitudes of wife beating, regarding violence as a notion of masculinity and the presence of strict gender roles in the society, were positively associated with IPV in pregnancy, and in concert with prior studies adressing these issue seperately [21, 28, 42]. Our study also supported that women reporting the presence of groups that legitimize men’s violence or accept wifebeating in their community, or report no social support for victims, are more likely to face increased risk of IPV in pregnancy [40, 43–45]. Women in these communities may share simillar attitudes and more readily accept IPV. In support of this finding, a recent qualitative study in Nepal stated that in a culture where violence is normalized and something endured, caused women to tolerate and accept the IPV situation [46]. Another study also confirmed that when couples report tolerant attitudes of IPV they are more likely to report spousal IPV [47].

Regarding individual related factors, this study confirmed that violence foster violence and witnessing IPV in childhood increases the likelihood of violent behaviour in the next generation [13, 23, 40, 42, 48]. Partner’s use of substance/s (alcohol, Khat, and/smoking), is a known and significant factor of IPV during pregnancy [4, 13, 14, 23, 40, 49–52], and enhancing if the partner also has mental health or personality problems [40, 53]. Factor related to the indivdual domain is the strongest in this model (Table 3), but with wide confidence intervals. This could be either due to small sample size, overlapping effect of the four factors or some other resons which requires further investigation. In general, our study supports the notion that an ecological model of IPV, as, a combination of societal, community, family and individual related factors, can help to explain the occurrence of IPV in pregnancy [21, 28, 54, 55]. The contribution of a combination of individual, relational, community and societal factors to the risk of IPV victimization has also been emphazised in WHO and CDC reports [40, 53].

Conflicting results exist about whether IPV increases or decreases during pregnancy [19, 56, 57]. In fact, our study showed that pregnancy neither stops nor prevents IPV. The prevalence estimates of IPV during pregnancy in this study are higher than previous studies undertaken in Ethiopia [11–15], and some other African countries [4, 17, 22, 30, 58–60]. However, other studies in Africa showed nearly similar or even higher prevalence [48, 57, 61]. A possible explanation for the high prevalence in our study could be the use of translated, internationally validated AAS tools and the data collectors strife at conducting the data collection in a safe environment that might have increased the rate of disclosure, or it could be a true increase in violence compared to prior Ethiopian studies. The contribution of keeping participants’ privacy and having a safe environment while obtaining data from sensitive issues, such as IPV, to increasing the disclosure rate has also been reported in other studies [24–27]. Ethiopia, being one of the highest IPV prevalent countries, the prevailing culture considering husband and wife issues as a private matter could thereby foster a community notion of having no obligation to intervene in family matters; all these may also explain the high prevalence of IPV reported in this study.

### Limitations and strength

Taking into account the complexity of factors impacting IPV in pregnancy, the simultaneous assessment of all social ecologic factors while controlling for potential confounders, the use of validated data collection tools, pretested and translated instruments to measure IPV in pregnancy, are some of the strengths of this study. The use of AIC and parsimony in selecting the best model, making inferences (good prediction) and interpret the result based on it may also lend strength.

The subject studied is very sensitive and even stigmatizing; assessment of IPV and its factors based on self-reporting might have introduced systematic errors (recall bias, non-disclosure, underreporting, and misclassification). Those who were exposed to IPV recently are more likely to report excessively, while those who were exposed a bit long ago may ignore or forget to report, which also might have introduced bias. Bias might have also been introduced due to unmeasured or poorly measured variables, confounders and interactions. The study had a cross sectional design (simultaneous collection of both exposure and outcome variables), and the setting is institutional based, we can neither claim causality nor generalizability, and calls for future prospective longitudinal study.

## Conclusion

Despite these limitations, this study provides valuable information and contributes to a better understanding of the magnitude of IPV in pregnancy and its multifaceted contributing factors in Ethiopia. The importance of community and societal factors in IPV warrant a public debate about IPV. Male involvement is imperative as impact of male norms related to and childhood experience of IPV are significant contributors to the risk of future IPV, as well as demonstrated in the literature, adverse events like chronic diseases and increased allostatic load [62]. As IPV also is a well-established risk factor for adverse pregnancy outcome [12, 17], the Ethiopian Ministry of Health could include IPV screening tools as an essential part of routine antenatal care. This study will also inform the efforts needed in achieving the new global health goals, specifically related to; [(Sustainable Development Goal] SDG target 3.1 and 3.2 (maternal and child survival); SDG target 5.2, (Eliminate all forms of violence against all women and girls), [63]]. Achieving these SDG targets require the attention of a range of professionals and agencies working in the area of clinical, epidemiological, anthropological, health policy and health systems.

In conclusion, our study, while maintaining that it is multi-factorial [28, 40], IPV in pregnancy is a common experience among women seeking antenatal care, and even more common than reported in many other studies, but within the range of other sub-Saharan African countries [4]. Given the high prevalence of IPV in Ethiopia, concurrent with high maternal and neonatal mortality rates [64, 65], the current study is relevant as the results from this study could contribute to the development of national policies or interventions to reduce pregnancy related IPV.

## Supporting information

S1 File REK Ethical clearance letter. (PDF)

S2 File The experience of intimate partner violence. (PDF)
