# Supplementary material for: Social ecological factors and intimate partner violence in pregnancy
Source: PLoS One. 2018 Mar 29;13(3):e0194681. doi: 10.1371/journal.pone.0194681 (PMC5875784; doi:10.1371/journal.pone.0194681)
Supplement: S1 File — (PDF) [file pone.0194681.s001.pdf]

Region:	Saksbehandler:	Telefon:	Vår dato:	Vår referanse:
REK nord			11.05.2015	2015/623/REK nord
			Deres dato:	Deres referanse:
			24.03.2015	

Vår referanse må oppgis ved alle henvendelser

Bosena Tebeje Gashaw
Jimma university

2015/623 Intimate Partner violence in Pregnancy: perceptions, magnitude, contributing factors an pregnancy outcomes

Forskningsansvarlig: Health and Society
Prosjektleder: Bosena Tebeje Gashaw

Vi viser til søknad om forhåndsgodkjenning av ovennevnte forskningsprosjekt. Søknaden ble behandlet av Regional komité for medisinsk og helsefaglig forskningsetikk (REK nord) i møtet 23.04.2015. Vurderingen er gjort med hjemmel i helseforskningsloven (hfl.) § 10, jf. forskningsetikkloven § 4.

Prosjektleders prosjektomtale
Background- Intimate partner violence (IPV) against women has increasingly been recognized in the international arena. However, the perceptions, magnitude of intimate partner violence and its impact in pregnancy outcome is not well explored in Ethiopia and not in Jimma town. The objective of this study is to assess Intimate Partner violence in pregnancy: perceptions, magnitude, contributing factors and pregnancy outcomes. Methods- Cross sectional and cohort study designs will be used in this study. A systematic as well as probability and purposive sampling method will be employed to obtain the required sample population for quantitative and qualitative study respectively. Pre-tested survey questionnaires, in-depth and focus group discussion guide will be used for data collection. Result and analysis- All quantitative data will be analyzed using SPSS version 20.0. The Qualitative data will be thematically analysed using Nvivo software.Ethical clearance will be secured from REK.

Vurdering

Dette er et PhD prosjekt ved universitetet i Oslo. Prosjektet skal i sin helhet gjennomføres i Etiopia og skal godkjennes der også. Studien er basert på informert samtykke. REK legger til grunn at prosjektet godkjennes i Etiopia.

Vedtak
Med hjemmel i helseforskningsloven §§ 2,9 10, samt forskningsetikkloven § 4 godkjennes prosjektet.

Sluttmelding og søknad om prosjektendring
Prosjektleder skal sende sluttmelding til REK nord på eget skjema senest 01.07.2018, jf. hfl. § 12. Prosjektleder skal sende søknad om prosjektendring til REK nord dersom det skal gjøres vesentlige endringer i forhold til de opplysninger som er gitt i søknaden, jf. hfl. § 11.

Klageadgang
Du kan klage på komiteens vedtak, jf. forvaltningsloven § 28 flg. Klagen sendes til REK nord. Klagefristen

er tre uker fra du mottar dette brevet. Dersom vedtaket opprettholdes av REK nord, sendes klagen videre til Den nasjonale forskningsetiske komité for medisin og helsefag for endelig vurdering.

Med vennlig hilsen

May Britt Rossvoll
sekretariatsleder

Kopi til: j.h.magnus@medisin.uio.no; postmottak@medisin.uio.no
