# Supplementary material for: Social ecological factors and intimate partner violence in pregnancy
Source: PLoS One. 2018 Mar 29;13(3):e0194681. doi: 10.1371/journal.pone.0194681 (PMC5875784; doi:10.1371/journal.pone.0194681)
Supplement: S2 File — (PDF) [file pone.0194681.s002.pdf]

The experience of Intimate Partner Violence: Abuse Assessment Screening tools (AAS).

1. Have you ever been (lifetime) emotionally or physically abused by your partner or someone important to you? Yes/No

2. Within the last one year, have you ever been hit, slapped, kicked, or otherwise physically hurted by some one? Yes/No

2.1. If Yes to Qn 2, who?

1. Husband	4. Stranger
2. Ex-Husband	5. In laws
3. Boyfriend	6. Multiple

3. Since you've been pregnant (current pregnancy), have you been slapped, kicked, or otherwise physically hurt by someone? Yes/No

3.1. If Yes to Qn 3, by who?

1. Husband	4. Stranger
2. Ex-Husband	5. In laws
3. Boyfriend	6. Multiple

3.2. If Yes to Qn 3 , where

1. On the face	4. Back
2. Head	5. Buttock
3. Abdomen	6.Other, state___

3.3. Score each incident according to the following scale:

1 = Threats of abuse including use of weapon

2 = Slapping, pushing; no injuries and/or lasting pain

3 = Punching, kicking, bruises, cuts and/or continuing pain

4 = Beating up, severe contusions, burns broken bones

5 = Head injury, internal injury, permanent Injury

6 = Use of weapon (gun, knife); wound from weapon

4. Within the last year, has anyone forced you to have sexual activities? Yes/No

4.1. If Yes to Qn 204, who?

1. Husband	4. Stranger
2. Ex-Husband	5. Multiple
3. Boyfriend	

5. Are you afraid of your partner or anyone listed above?

6. Within the last year have you been insulted, belittled, constantly humiliated, intimidated (e.g. destroying things), threatened of being harmed, threatened to take away your children) by your partner/husband? Yes/No

7. Within the last year have you have been controlled by your partner? Yes/No

7.1 .If Yes to Qn 7, which type of controlling imposed on you by your partner?

1. Isolating you from your family and friends
2. Monitoring your movements
3. Restricting you to access to financial resources
4. Restricting you from employment, education or medical care

8. Where did you turn in the incidents of any IPV?

1. Your family
2. Your neighbours
3. Your religious father
4. Keep silent
5. Other, state____

8.1 If you keep silent, state major reason/s__________
